# Supplementary material for: Continuity of care in general practice in Norway
Source: PLoS One. 2024 Jul 11;19(7):e0305164. doi: 10.1371/journal.pone.0305164 (PMC11238959; doi:10.1371/journal.pone.0305164)
Supplement: S1 Appendix — (DOCX) [file pone.0305164.s001.docx]

# Appendix

S1 Table: Number of patients and physicians

|  | **Number of patients with at least one consultation** | **Number of physicians with at least one consultation** |
| --- | --- | --- |
| **2006** | 3163070 | 6489 |
| **2007** | 3215465 | 6728 |
| **2008** | 3294315 | 6976 |
| **2009** | 3342160 | 7175 |
| **2010** | 3431985 | 7270 |
| **2011** | 3538693 | 7511 |
| **2012** | 3557812 | 7628 |
| **2013** | 3566931 | 7761 |
| **2014** | 3622285 | 7751 |
| **2015** | 3650764 | 7836 |
| **2016** | 3700313 | 8140 |
| **2017** | 3741715 | 8324 |
| **2018** | 3755807 | 8479 |
| **2019** | 3782975 | 8652 |
| **2020** | 3730602 | 8958 |
| **2021** | 3838052 | 9092 |

S2 Table: St. Leonard’s Index of Continuity of Care (SLICC)

|  | **Nationally** | | **Highly central** | | **Moderately central** | | **Least central** | |
| --- | --- | --- | --- | --- | --- | --- | --- | --- |
|  | Proportion | Sd | Proportion | Sd | Proportion | Sd | Proportion | Sd |
| **2006** | 62,5 | 0,01 | 66,5 | 0,02 | 63,8 | 0,02 | 47,3 | 0,04 |
| **2007** | 62,6 | 0,01 | 66,1 | 0,02 | 63,8 | 0,02 | 48,7 | 0,04 |
| **2008** | 62,3 | 0,01 | 65,7 | 0,02 | 63,3 | 0,02 | 49,3 | 0,04 |
| **2009** | 62,5 | 0,01 | 65,2 | 0,02 | 64,1 | 0,02 | 49,3 | 0,04 |
| **2010** | 61,7 | 0,01 | 65,4 | 0,02 | 62,5 | 0,02 | 48,2 | 0,04 |
| **2011** | 61,4 | 0,01 | 65,0 | 0,02 | 62,4 | 0,02 | 47,0 | 0,04 |
| **2012** | 61,5 | 0,01 | 65,0 | 0,02 | 62,8 | 0,02 | 47,1 | 0,04 |
| **2013** | 61,1 | 0,01 | 63,9 | 0,02 | 62,6 | 0,02 | 47,4 | 0,04 |
| **2014** | 61,3 | 0,01 | 64,9 | 0,02 | 62,1 | 0,02 | 47,1 | 0,04 |
| **2015** | 61,6 | 0,01 | 65,2 | 0,02 | 62,7 | 0,02 | 47,2 | 0,04 |
| **2016** | 61,7 | 0,01 | 65,3 | 0,02 | 62,2 | 0,02 | 48,1 | 0,04 |
| **2017** | 61,5 | 0,01 | 65,1 | 0,02 | 62,1 | 0,02 | 47,8 | 0,04 |
| **2018** | 61,2 | 0,01 | 64,4 | 0,02 | 61,7 | 0,02 | 48,9 | 0,04 |
| **2019** | 61,5 | 0,01 | 64,2 | 0,02 | 62,1 | 0,02 | 50,0 | 0,04 |
| **2020** | 63,9 | 0,01 | 66,9 | 0,02 | 64,5 | 0,02 | 50,2 | 0,04 |
| **2021** | 64,4 | 0,01 | 68,1 | 0,02 | 64,6 | 0,02 | 49,4 | 0,04 |

S3 Table: The Usual Provider of Care (UPC) Index

|  | **Nationally** | | **Highly central** | | **Moderately central** | | **Least central** | |
| --- | --- | --- | --- | --- | --- | --- | --- | --- |
|  | Proportion | Sd | Proportion | Sd | Proportion | Sd | Proportion | Sd |
| **2006** | 71,1 | 0,01 | 73,9 | 0,02 | 71,6 | 0,02 | 61,8 | 0,04 |
| **2007** | 70,4 | 0,01 | 73,4 | 0,02 | 70,8 | 0,02 | 60,8 | 0,04 |
| **2008** | 69,4 | 0,01 | 72,1 | 0,02 | 69,7 | 0,02 | 60,2 | 0,04 |
| **2009** | 69,0 | 0,01 | 71,5 | 0,02 | 69,8 | 0,02 | 59,2 | 0,04 |
| **2010** | 67,9 | 0,01 | 70,9 | 0,02 | 68,3 | 0,02 | 58,0 | 0,04 |
| **2011** | 67,3 | 0,01 | 70,6 | 0,02 | 67,6 | 0,02 | 56,5 | 0,04 |
| **2012** | 67,2 | 0,01 | 70,1 | 0,02 | 67,8 | 0,02 | 56,5 | 0,03 |
| **2013** | 66,7 | 0,01 | 69,0 | 0,02 | 67,5 | 0,02 | 56,6 | 0,04 |
| **2014** | 66,7 | 0,01 | 69,7 | 0,02 | 67,1 | 0,02 | 56,2 | 0,04 |
| **2015** | 67,0 | 0,01 | 69,9 | 0,02 | 67,6 | 0,02 | 56,0 | 0,04 |
| **2016** | 66,9 | 0,01 | 69,8 | 0,02 | 67,0 | 0,02 | 57,0 | 0,04 |
| **2017** | 66,9 | 0,01 | 69,8 | 0,02 | 67,0 | 0,02 | 56,9 | 0,03 |
| **2018** | 66,6 | 0,01 | 69,2 | 0,02 | 66,6 | 0,02 | 58,1 | 0,04 |
| **2019** | 67,2 | 0,01 | 69,4 | 0,02 | 67,6 | 0,02 | 58,7 | 0,03 |
| **2020** | 70,1 | 0,01 | 72,6 | 0,02 | 70,5 | 0,02 | 59,5 | 0,04 |
| **2021** | 71,4 | 0,01 | 74,2 | 0,02 | 71,3 | 0,02 | 61,1 | 0,03 |
